# Editorial: Biological and non-pharmacological treatments of obsessive-compulsive disorder and related disorders

**DOI:** 10.3389/fpsyt.2024.1494444

**Published:** 2024-10-22

**Authors:** Renato de Filippis, Samer El Hayek, Mohammadreza Shalbafan

**Affiliations:** ^1^ Psychiatry Unit, Department of Health Sciences, University Magna Graecia of Catanzaro, Catanzaro, Italy; ^2^ Medical Department, Erada Center for Treatment and Rehabilitation in Dubai, Dubai, United Arab Emirates; ^3^ Mental Health Research Center, Psychosocial Health Research Institute, Department of Psychiatry, School of Medicine, Iran University of Medical Sciences, Tehran, Iran; ^4^ Brain and Cognition Clinic, Institute for Cognitive Sciences Studies, Tehran, Iran

**Keywords:** corticospinal excitability, deep brain stimulation, mental health, obsessive-compulsive disorder, obsessive behavior, psychiatry, treatment-resistant obsessive-compulsive disorder

Pharmacological treatments for mental disorders, while often effective, can sometimes have poor or absent clinical responses (1). This includes cases of resistance in both acute and chronic forms of illnesses, with or without severe comorbidities. Additionally, psychosocial interventions may be limited in their effectiveness, availability, and scalability (2, 3).

In recent years, several non-pharmacological biological treatments have become increasingly available (4). Such neuromodulatory interventions, which involve direct electro-/electromagnetic stimulation of targeted brain areas, include invasive options like deep brain stimulation (DBS) and vagus nerve stimulation (VNS), as well as minimally invasive approaches such as transcranial magnetic stimulation (TMS) and its variants, transcranial direct current stimulation (tDCS), electroconvulsive therapy (ECT), light therapy (LT), and other interventions as sleep deprivation (5). These therapies have been applied as standalone treatments or in combination with medications for various psychiatric conditions, including mood disorders, schizophrenia, and obsessive-compulsive disorder (OCD) (6–8). The latter is one of the major psychiatric illnesses, with an early onset and a high impact on quality of life and functioning of patients (9, 10).

This Research Topic collection, titled “*Biological and Non-Pharmacological Treatments of Obsessive-Compulsive Disorder and Related Disorders”*, comprises seven articles that address updates in OCD management from both treatment and social perspectives.

To start with, Rodrigues da Silva et al. performed a systematic review and meta-analysis of case-control studies comparing various measures of motor cortical excitability, including after repetitive TMS (rTMS) treatment, in patients with OCD compared to healthy individuals. Based on their findings, there is an inhibitory deficit in motor cortex excitability in patients with OCD compared to controls. Regardless of its impact on OCD symptoms, rTMS does not appear to alter resting motor threshold, possibly because this measure reflects glutamatergic synaptic transmission, while rTMS is primarily believed to affect GABAergic function. This review highlights the importance of continuing research into OCD pathophysiology using the cortical silent period and other accessible, non-invasive measures of cortical excitability.

Abdelnaim et al. conducted a study using DBS in patients with severe, chronic, treatment-resistant OCD. Eleven patients underwent bilateral DBS implantation in the bed nucleus of the stria terminalis (BNST) and were evaluated using the Yale-Brown Obsessive-Compulsive Scale (Y-BOCS), among other scales, with a one-year follow-up after optimized stimulation. Overall, the mean Y-BOCS score decreased from 28.3 at baseline to 13.3 at the last follow-up, representing a 53% reduction. This improvement in OCD symptoms was accompanied by decrease in depressive symptoms and amelioration in global functioning and quality of life. These findings suggest that BNST-DBS can be effective for patients with treatment-resistant OCD, leading to symptom reduction and overall improvement in functioning.

In the realm of emerging treatments, Ching et al. designed a study protocol for a randomized, double-blind, placebo-controlled, non-crossover design to investigate the clinical and neural impacts of a single dose of oral psilocybin (0.25 mg/kg) compared to an active placebo control (250 mg of niacin) on OCD symptoms. They will enroll 30 adult participants at a single site in Connecticut, USA, all of whom have not responded to at least one standard treatment (medication or psychotherapy) for OCD. Participants will be assessed using the Y-BOCS and Visual Analog Scale, with a total follow-up period of 12 weeks. Resting-state neuroimaging data will be collected at baseline and at the primary endpoint. This study could represent a significant step forward in treating refractory OCD and may pave the way for future research into the neurobiological mechanisms of OCD that could respond to psilocybin treatment.

Zengil and Laloğlu conducted a clinical study involving 60 patients diagnosed with OCD and 30 healthy controls. They used the Y-BOCS and Hamilton Depression Rating Scale (HDRS) to assess the OCD group. The results showed that levels of zonulin and occludin were significantly higher in the OCD group compared to the control group (p<0.001). Additionally, these biomarkers were significantly elevated in patients with OCD and comorbid major depressive disorder (MDD) compared to those without MDD (p<0.001). A positive correlation was found between the duration of OCD and the levels of zonulin and occludin. Furthermore, in the OCD group, there was a moderate positive correlation between Y-BOCS and HDRS scores and the levels of these biomarkers. In summary, this study found significantly higher levels of zonulin and occludin in patients with OCD compared to healthy controls, with these increases correlating positively with disease duration and severity. The elevation was more pronounced in patients with comorbid MDD. These findings suggest a potential disruption of the intestinal and blood-brain barriers in patients with OCD.

In their systematic review, Eghdami et al. evaluated the safety and effectiveness of N-acetylcysteine (NAC), a glutamate-modulating agent, as an augmentation therapy for moderate-to-severe OCD. The primary outcome was the mean difference in Y-BOCS scores before and after NAC augmentation. No significant differences were found for treatment durations shorter than five weeks or longer than 12 weeks. Similarly, there were no significant differences between the experimental and control groups regarding obsession and compulsion subscale scores on the Y-BOCS. In terms of adverse events, no significant differences were observed between the two groups. The results of this study indicated a positive outcome for the experimental group in terms of the total Y-BOCS score when using the medication for a period of five to eight weeks.

The article by Weiss et al. examined the relationship between context and obsessions through a narrative review. The authors summarized current evidence on how context distinguishes obsessions in OCD from intrusive thoughts in both affected and unaffected individuals. According to the authors, novel research suggests that the cognitive processes underlying obsessions may significantly differ in patients with OCD from those in healthy individuals, particularly regarding their relationship to context. The review covers five studies: two involving individuals diagnosed with OCD and three exploring the relationship between OCD symptoms and context in unaffected individuals. Based primarily on self-reported data, the review investigates the connection between thoughts and their context, highlighting how the repetition and automaticity of thoughts, as well as their detachment from context over time, contribute to defining obsessions as distinct from intrusive thoughts. Future research should aim to confirm this hypothesis through experimental evidence and determine whether this shift is a cause or consequence of the disorder.

Finally, the article by Hassan et al. explored the variations in OCD symptomatology across cultural dimensions. The authors emphasized the impact of the socio-cultural context on the genesis, severity, and interpretation of obsessive symptoms and thoughts. They argue that these symptoms are often underestimated or overestimated because they are not considered within the context of the patient’s culture. Therefore, it is essential for clinicians to account for the patient’s sociocultural context when addressing OCD and mental health in general. The authors asserted that culturally competent clinical practice is not only an ethical obligation but also crucial for delivering effective, patient-centered care to individuals from diverse backgrounds.

In conclusion, there is a pressing need for further research in this area. While progress has been made in recognizing the role of new treatments for OCD, much work remains. The growing body of knowledge on the relationship between OCD and various biological non-pharmacological treatments should continue to expand, with future research focusing on specific treatment management and protocols. Moreover, research should aim to develop interventions and treatment strategies that respect cultural values and beliefs while providing evidence-based care (11–13).

## References

[1] de Filippis R De Fazio P Gaetano R Steardo L Cedro C Bruno A. Current and emerging long-acting antipsychotics for the treatment of schizophrenia. Expert Opin Drug Saf. (2021) 20:771–90. doi: 10.1080/14740338.2021.1910674 33775184

[2] Bighelli I Rodolico A García-Mieres H Pitschel-Walz G Hansen W-P Schneider-Thoma J. Psychosocial and psychological interventions for relapse prevention in schizophrenia: a systematic review and network meta-analysis. Lancet Psychiatry. (2021) 8:969–80. doi: 10.1016/S2215-0366(21)00243-1 34653393

[3] Bhardwaj A Gurung D Rai S Kaiser BN Cafaro CL Sikkema KJ. Treatment Preferences for Pharmacological versus Psychological Interventions among Primary Care Providers in Nepal: Mixed Methods Analysis of a Pilot Cluster Randomized Controlled Trial. Int J Environ Res Public Health. (2022) 19:2149. doi: 10.3390/ijerph19042149 35206331 PMC8871897

[4] Brunoni AR Sampaio-Junior B Moffa AH Aparício LV Gordon P Klein I. Noninvasive brain stimulation in psychiatric disorders: a primer. Braz J Psychiatry. (2019) 41:70–81. doi: 10.1590/1516-4446-2017-0018 30328957 PMC6781710

[5] Rosson S de Filippis R Croatto G Collantoni E Pallottino S Guinart D. Brain stimulation and other biological non-pharmacological interventions in mental disorders: an umbrella review. Neurosci Biobehav Rev. (2022) 139:104743. doi: 10.1016/j.neubiorev.2022.104743 35714757

[6] de Filippis R Aguglia A Costanza A Benatti B Placenti V Vai E. Obsessive–compulsive disorder as an epiphenomenon of comorbid bipolar disorder? An updated systematic review. J Clin Med. (2024) 13:1230. doi: 10.3390/jcm13051230 38592113 PMC10931838

[7] Razza LB Palumbo P Moffa AH Carvalho AF Solmi M Loo CK. A systematic review and meta-analysis on the effects of transcranial direct current stimulation in depressive episodes. Depress Anxiety. (2020) 37:594–608. doi: 10.1002/da.23004 32101631

[8] Mutz J Vipulananthan V Carter B Hurlemann R Fu CHY Young AH. Comparative efficacy and acceptability of non-surgical brain stimulation for the acute treatment of major depressive episodes in adults: systematic review and network meta-analysis. BMJ. (2019) 364:l1079. doi: 10.1136/bmj.l1079 30917990 PMC6435996

[9] Gaetano R de Filippis R Segura-Garcia C De Fazio P. Impact of Bipolar Disorder and Obsessive-Comulsive Dioserder comorbidity on neurocognitive profile: a mini-review. Psychiatr Danub. (2020) 32:346–50. doi: 10.24869/psyd.2020.346 33370731

[10] de Filippis R Aloi M Bruni A Gaetano R Segura-Garcia C De Fazio P. Bipolar disorder and obsessive compulsive disorder: The comorbidity does not further impair the neurocognitive profile. J Affect Disord. (2018) 235:1–6. doi: 10.1016/j.jad.2018.03.010 29627704

[11] Shalbafan M Malekpour F Tadayon Najafabadi B Ghamari K Dastgheib S-A Mowla A. Fluvoxamine combination therapy with tropisetron for obsessive-compulsive disorder patients: A placebo-controlled, randomized clinical trial. J Psychopharmacol. (2019) 33:1407–14. doi: 10.1177/0269881119878177 31575326

[12] Hadi F Kashefinejad S Kamalzadeh L Hoobehfekr S Shalbafan M. Glutamatergic medications as adjunctive therapy for moderate to severe obsessive-compulsive disorder in adults: a systematic review and meta-analysis. BMC Pharmacol Toxicol. (2021) 22:69. doi: 10.1186/s40360-021-00534-6 34736541 PMC8569963

[13] Askari S Mokhtari S Shariat SV Shariati B Yarahmadi M Shalbafan M. Memantine augmentation of sertraline in the treatment of symptoms and executive function among patients with obsessive-compulsive disorder: A double-blind placebo-controlled, randomized clinical trial. BMC Psychiatry. (2022) 22:34. doi: 10.1186/s12888-021-03642-z 35022014 PMC8753835

